# Sensor Localization from Distance and Orientation Constraints

**DOI:** 10.3390/s16071096

**Published:** 2016-07-15

**Authors:** Josep M. Porta, Aleix Rull, Federico Thomas

**Affiliations:** Institut de Robòtica i Informàtica Industrial, CSIC-UPC, Llorens Artigas 4-6, Barcelona 08028, Spain; arull@iri.upc.edu (A.R.); thomas@iri.upc.edu (F.T.)

**Keywords:** localization, sensor networks, distance constraints, orientation constraints, Distance Geometry

## Abstract

The sensor localization problem can be formalized using distance and orientation constraints, typically in 3D. Local methods can be used to refine an initial location estimation, but in many cases such estimation is not available and a method able to determine all the feasible solutions from scratch is necessary. Unfortunately, existing methods able to find all the solutions in distance space can not take into account orientations, or they can only deal with one- or two-dimensional problems and their extension to 3D is troublesome. This paper presents a method that addresses these issues. The proposed approach iteratively projects the problem to decrease its dimension, then reduces the ranges of the variable distances, and back-projects the result to the original dimension, to obtain a tighter approximation of the feasible sensor locations. This paper extends previous works introducing accurate range reduction procedures which effectively integrate the orientation constraints. The mutual localization of a fleet of robots carrying sensors and the position analysis of a sensor moved by a parallel manipulator are used to validate the approach.

## 1. Introduction

The accurate localization of a sensor is fundamental in many applications. For instance, one of the main problems to address in sensor networks is the location of the nodes since most of the objectives of such networks rely on the correct association between the sensor readings and the location information [1]. Moreover, the position of the nodes can be exploited to optimize aspects such as the power consumption of the network [2]. Even for isolated sensors, the position at which each measure is taken is fundamental to determine, for instance, the exact place where there is a defect in a pipe probed with a radar [3] or a problem in an electronic device inspected with ultrasounds [4].

Sensor localization is typically based on angular and distance information. Distance Geometry studies problems characterized by distance and orientation constraints between a given set of points [5]. It provides a formulation invariant to the reference frame and, therefore, it gives geometric insights on different problems that remain concealed when using Cartesian geometry. These insights allow deriving solutions common to problems that otherwise have to be treated on a case-by-case basis [6]. Due to its generality, Distance Geometry finds application in many fields, including biochemistry [7], nano-technology [8], inverse/direct kinematics of serial/parallel robots [9], singularity analysis [10], or fusion of sensor data [11]. This paper proposes novel Distance Geometry tools for the sensor localization problem.

Arguably, the main issue of existing Distance Geometry techniques is that they can not deal with orientation constraints, which are fundamental in many applications [12,13]. The common alternative is to first take into account only the distance constraints to generate all the possible solutions and then rule out those with wrong orientations in a post-processing. However, in principle, the orientation constraints can be integrated into the solver and exploited to focus the search effort, thus increasing the computational efficiency. To the best of our knowledge, only two approaches exists that combine distance and orientation constraint [14,15], but they are limited to 1D or 2D problems, respectively, and their possible extensions to solve higher-dimensional problems is non-feasible.

This paper presents an alternative that naturally scales to higher dimensions. The proposed procedure is geometric, avoiding the use of Cayley-Menger determinants, the dominant tool in Distance Geometry [16,17,18], which generates formulations that rapidly get involved with the number of points and their dimension [19,20]. The method extends some of the ideas in [21], which implements geometric methods to project the problem to a lower dimensional sub-space, reduce the feasibility ranges for the variable distances in the problem, and back-project these reduced ranges to the original dimension to obtain a tighter approximation of the solution set. Herein, the approach in [21] is improved by introducing novel projection and back-projection methods that clearly outperform the original ones. Moreover, the range reduction procedure proposed in this paper effectively integrates the orientation constraints, which were not considered in [21].

This paper is structured as follows. Next section frames the proposed approach in the context of existing works and Section 3 formalizes the problem addressed in this paper. Then, Section 4 describes the projection, range reduction, and back-projection procedures, and Section 5 experimentally evaluates them. Finally, Section 6 summarizes the contributions of this work and points to issues deserving further consideration.

## 2. Related Work

The problem of localization appears in many fields, including sensor networks, mobile robotics, or in the position analysis of mechanisms. In all the cases, the problem is to localize one or more devices from sensor information. The sensor information can be based on distances (either given by the configuration of the sensing device or computed from time of flight or from signal strength) [22,23,24], on angles [25,26,27], or even on signal profiling [28,29,30]. The latter approach can produce accurate results, but it requires a map of the signal profile acquired offline, which is not available in general. Thus, the method introduced in this paper addresses the sensor localization problem using only distance and angular information acquired on-line, but it is agnostic on how the distance and orientation constraints are obtained. Actually, the angular information required in the proposed method is just the relative orientation of particular point sets, which is easier to obtain than accurate angular measures.

In any case, the sensor readings will be affected by the inaccuracies inherent to any physical process. The way in which such inaccuracies are modeled characterizes the two main families of approaches to the localization problem. The first one, which is the most popular, represents the inaccuracies with probabilistic models [31,32,33,34,35,36,37,38,39,40]. In this case, maximum likelihood estimators are typically used to determine the most probable solution to the problem using iterative procedures that require an initial point relatively close to the optimal solution. Such initialization points are not easy to determine. Moreover, as remarked in [41] (p. 2549), the probabilistic approaches assume unbiased distributions, which is a hypothesis hard to validate in practice due to the existence of flex and flip ambiguities [42]. For this reason, set theoretic approaches have been proposed, where the sensor inaccuracies are bounded in given intervals, but where not assumption is taken about the error distribution inside such ranges [43]. The method proposed in this paper belongs to this second family of approaches.

The space where the problem is formalized gives another broad classification of localization methods. In this case, the dominant approach formalizes the sensor localization in Cartesian space [24,44,45,46,47,48] introducing arbitrary reference frames, anchor nodes, and non-linearities when transforming distances to coordinates. A better alternative, explored herein, is to reduce the location uncertainty directly in distance space and compute coordinates only when uncertainties are reduced using a process of error propagation [18,42,49,50,51,52]. Moreover, like the method described in [53], our approach does not require anchor nodes for the range reduction since they are only used, if available, when computing coordinates.

A popular approach operating in distance space is Multidimensional Scaling (MDS) [49]. In this approach, an initial estimation for the missing distances is refined by projecting the distance matrix to the cone of positive semidefinite matrices to obtain the closer valid distance matrix in the least square sense. In related approaches, semidefinite programming [50,51,54,55] and simulated annealing [42,47] are used to obtain better approximations in the cone of semidefinite matrices and to avoid local minima. Such approaches assume a probabilistic model of the error, operate on points, can not guarantee the final solution to be inside pre-define ranges, and only provide good results if the number of known distances is relatively large. In the extreme case in which enough distances are initially known, trilateration methods can be used to determine the location of the sensors using a greedy procedure [11,20,52,56]. While the methods that project to the cone of semidefinite matrices only provide one solution to the problem, trilateration-based methods can deliver all the valid solutions. Actually, the determination of all the valid localizations is a fundamental problem in distance-based sensor localization [41]. The approach presented in this paper is related to the MDS method in the sense that it also decomposes a given matrix to determine valid coordinates for the sensors to localize. However, our approach adopts a set theoretic approach which guarantees the solutions to be inside the given ranges and, like in the trilateration approaches, it determines all the valid solutions for a given localization problem. Additionally, a relevant distinctive feature of the approach is that it can incorporate orientation constraints to avoid flip ambiguities, focusing only on the valid solutions. To the best of our knowledge, no other distance-based approaches exist able to integrate the orientation constraints in the search process for arbitrary dimensional problems. Moreover, the method is also able to deal with flex ambiguities providing a complete approximation of the set of valid locations if such ambiguities occur.

Finally, in sensor networks there is a difference between centralized and distributed localization systems [38,57,58]. In principle, the latter offer better scalability and they are energetically more efficient, but they must be carefully designed to ensure convergence and to control the error propagation. While the approach introduced in this paper can be combined with distributed approaches that fuse local position estimations [59], we describe it as a centralized procedure to simplify the presentation.

Summarizing, this paper presents a sensor localization system able to integrate distance and orientation information, which operates in distance space to avoid non-linearities and arbitrary reference frames, without making any assumption on the distribution of errors in the input data, and that is able to determine all the solutions of the given problem without any initial estimation, even if the solution set is a flex. All these features make the proposed system a unique approach in the field.

## 3. Problem Formalization

The general problem addressed here can be formalized as follows. Given $s_{i,j}$, with 1≤i,j≤n, the square of $d_{i,j}$, the distance between points $P_{i}$ and $P_{j}$ in $R^{q}$, and $\sigma_{F,i,k}$, the desired relative orientation between any two *q*-dimensional simplices sharing hyperface *F* defined by $\left\{P_{i_{1}},\ldots ,P_{i_{q}}\right\}$, and with apexes at $P_{i}$ and $P_{k}$, the objective is to find all the possible coordinates for the points up to rigid transformations, such that the given distance and orientation constraints are fulfilled. Typically, *q* is 2 or 3 and, by convention, $\sigma_{F,i,k}$ is set to −1 if the two simplices must be on the same side of the hyperplane defined by *F*, +1 if they have to be on opposite sides of this hyperplane, and 0 if their relative orientation is not fixed.

Since rigid transformations have to be disregarded, the coordinates of one of the points, say $P_{1}$, can be arbitrarily fixed to $x_{1}$ and the rest of the yet unknown coordinates can be arranged in a (n−1)×q matrix

(1) $$X=\left(\begin{array}{c} x_{2}-x_{1}\\ x_{3}-x_{1}\\ \ldots \\ x_{n}-x_{1} \end{array}\right)$$

Then, the (n−1)×(n−1) matrix

(2) $$G=X\;X^{\;⊤}$$

is known as the Gram matrix. If all the squared inter-point distances are know, the entries of this matrix can be computed using the cosine law, without resorting to the coordinates

(3) $$G_{i,j}=\left(x_{i}-x_{1}\right)\;\;{\left(x_{j}-x_{1}\right)}^{\;⊤}=d_{1,i}\;d_{1,j}\;\cos\left(\alpha \right)=\frac{1}{2}\;\left(s_{1,i}+s_{1,j}-s_{i,j}\right)$$

with *α* the angle between $\vec{P_{1}P_{i}}$ and $\vec{P_{1}P_{j}}$.

Schoenberg showed that a set of inter-point distances can be embedded in $R^{q}$ if and only if G is a positive semidefinite matrix with range *q* [60]. In this case, the first *q* steps of the Cholesky factorization of G are feasible and generate X, from which the desired coordinates can be readily determined [16]. Thus, in the context of Distance Geometry, the Cholesky factorization can be interpreted as a process of determining the *q*-dimensional coordinates of a set of points from their pair-wise distances. The first step of the factorization gives the first coordinate for all the points (i.e., the first column of X) and defines a set of distances in a q−1 dimensional space that, once recursively processed, provides the remaining q−1 columns of X.

Thus, if all the pair-wise distances are know, the Cholesky factorization yields the desired set of coordinates. However, due to characteristics of the problem or to sensor range limitations, in general only few of the input distances are know [61]. The rest of them are corrupted with noise and systematic errors, which are assumed bounded, but without any particular noise probability distribution inside the bounds [43]. Thus, distances may be exactly known (in which case the initial lower and upper bounds coincide), unknown (in which case the initial range goes from zero to infinity), or estimated using sensor information (in which case an initial finite range is known). The Cartesian product of such ranges defines an initial box, i.e., an orthotope, in distance space which includes the solutions for the problem at hand.

**Figure 1:**
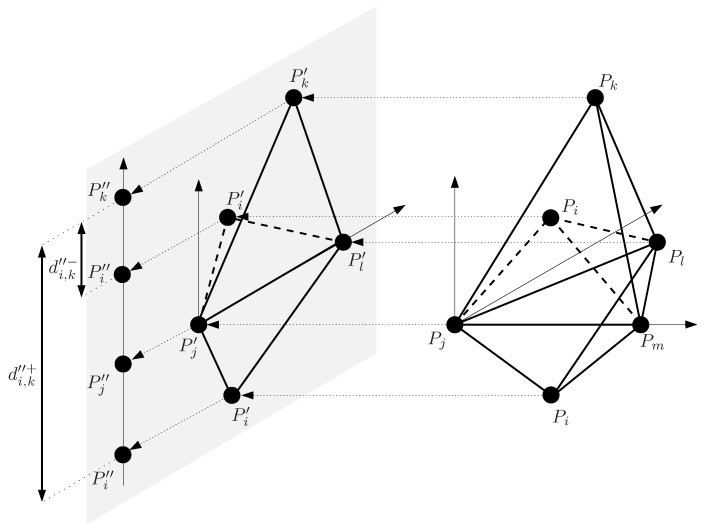
Schematic illustration of the projection and back-projection range reduction procedure proposed in this paper. See the text for details.

**Figure 2:**
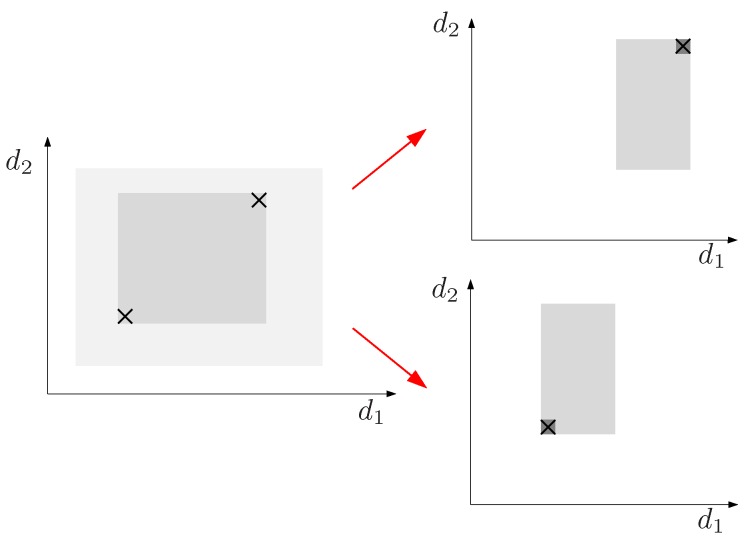
Searching for two solutions (indicated by crosses) in a two-dimensional distance space using a branch-and-prune approach. The initial search space (light gray rectangle on the left plot) is reduced as much as possible, but without leaving out any solution. The result is the middle-gray rectangle. Then this space is bisected and the search is repeated independently in the two resulting sub-spaces, as shown in the right part of the figure. Finally, the solutions are isolated as small boxes in distance space (the dark-gray squares around the crosses).

The approach proposed here to find all the solutions in the given initial box is based on the recursive Cholesky factorization and it is illustrated in Figure 1. Let’s suppose that we have five points, $P_{i}$, $P_{j}$, $P_{k}$, $P_{l}$, and $P_{m}$, and that all the distances between them are known, except $d_{i,k}$. Thus, in this example, the initial search space is $d_{i,k}\in \left[0,+\infty \right]$, i.e., a one-dimensional box. Fixing $P_{j}$, $P_{l}$, $P_{m}$ and $P_{k}$, point $P_{i}$ can be placed at two locations relative to the plane defined by $P_{j}$, $P_{l}$, and $P_{m}$. The approach proposed in this paper first projects the points to a plane orthogonal to $\bar{P_{j}P_{m}}$ to obtain $P_{i}^{\prime }$, $P_{j}^{\prime }$, $P_{k}^{\prime }$, and $P_{l}^{\prime }$. Then, these points are projected to a line orthogonal to $\bar{P_{j}^{\prime }P_{l}^{\prime }}$ to obtain $P_{i}^{\prime \prime }$, $P_{j}^{\prime \prime }$, and $P_{k}^{\prime \prime }$. Actually, the projections are not explicitly computed, but the distance between projected points is directly obtained. Two possible values exist for the distance between $P_{i}^{\prime \prime }$ and $P_{k}^{\prime \prime }$, $d_{i,k}^{\prime \prime -}$ and $d_{i,k}^{\prime \prime +}$. If no orientation constraint is introduced in this problem the range for this distance must include these two values, i.e., $d_{i,k}^{\prime \prime }\in \left[d_{i,k}^{\prime \prime -},d_{i,k}^{\prime \prime +}\right]$. This range once back-projected (i.e., when undoing the projection steps) gives a range for $d_{i,k}$, $\left[d_{i,k}^{-},d_{i,k}^{+}\right]$, tighter than the initial search box. This range can not be further reduced by projection and back-projection and, thus, to isolate the two solutions of this problem the branch-and-bound approach illustrated in Figure 2 is adopted. The obtained range is split in two sub-ranges, $\left[d_{i,k}^{-},c\right]$ and $\left[c,d_{i,k}^{+}\right]$ with $c=(d_{i,k}^{-}+d_{i,k}^{+})/2$, which are processed independently to isolate the two solutions. However, if an orientation constraint is introduced indicating, for instance, that $P_{i}$ and $P_{k}$ must be at opposite sides of the plane defined by $P_{j}$, $P_{l}$, and $P_{m}$, only one of the two solutions is valid. This solution can be directly identified in a single iteration and without any range division fixing $d_{i,k}^{\prime \prime }$ to $d_{i,k}^{\prime \prime +}$, which once back-projected gives a single value for $d_{i,k}$.

Schoenberg’s characterization of feasibility has been exploited projecting incomplete distance matrices to the cone of semidefinite matrices with the aim of determining one valid set of coordinates [49,50,51,54], while we are interested in all the possible ones in the given input intervals. Cholesky methods for interval matrices exist and might be used [62,63], but they assume a Gram matrix with uncorrelated entries, which is not our case, as it can be concluded from Equation (3) since several entries of G involve the same squared distances. These correlations induce overestimations in interval arithmetics [64]. Consider, for instance, that we want to determine the range of

(4) $$f\left(x\right)=x^{2}-x$$

with x∈[−1,1]. Plain interval arithmetics evaluates *f* as if its two terms were independent

(5) $$f\left(x\right)\in {\left[-1,1\right]}^{2}-\left[-1,1\right]=\left[0,1\right]-\left[-1,1\right]=\left[-1,2\right]$$

while the actual range of *f* in the given domain is [−0.25,2]. The method presented in [21] interprets the Cholesky factorization geometrically to deal with distance constraints, but it is based on interval arithmetics and, thus, it is also affected by the overestimation issue.

In the approach presented in this paper, the correlations are taken into account to provide tighter range evaluations and, thus, a faster convergence to the solutions. Note, however, that since we are working with ranges and not with points Schoenberg’s approach only provides necessary conditions for realizability. Thus, the method presented next is based on necessary conditions that progressively isolate the solution set.

## 4. Isolating the Solution Set via Projections and Back-Projections

Next, we describe the three basic steps of the proposed method: projection on a given hyperplane, distance range reduction, and back-projection. Projections on different hyperplanes can be used to reduce the ranges and, when no further reduction is possible, the largest distance range is split at its central point and the two resulting sub-problems are treated independently. This process is iterated until the solution set is isolated with a desired accuracy. In this process some empty boxes, i.e., boxes resulting from a bisection which include no solutions, may be generated. The performance of a branch-and-prune solver can be evaluated by the number of empty boxes generated, i.e., the lower the better since the ideal algorithm would converge directly to the solutions without exploring empty boxes.

### 4.1. Projection

**Figure 3:**
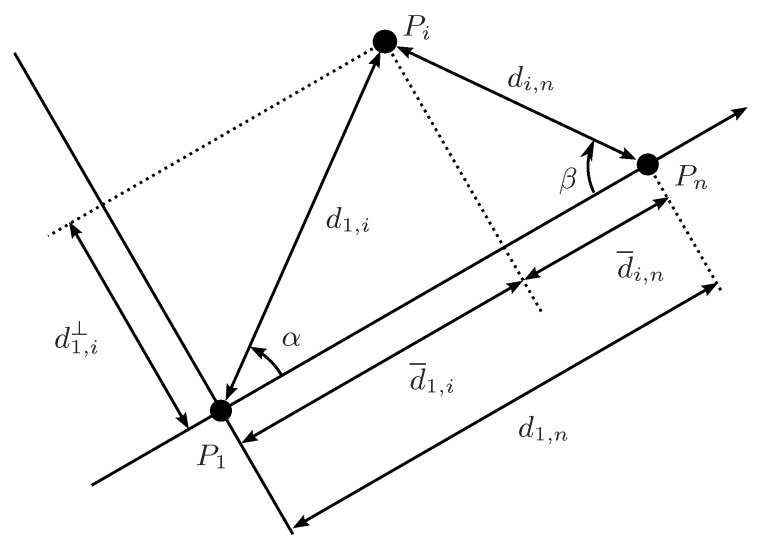
Projecting distances on the axis defined by $\bar{P_{1}P_{n}}$ and on its orthogonal complement.

Figure 3, illustrates a single step of the Cholesky factorization of the Gramian associated with a distance matrix. For instance, the element $d_{1,i}=\sqrt{s_{1,i}}$ is decomposed into ${\bar{d}}_{1,i}$, the projection of $d_{1,i}$ on the axis defined by $\bar{P_{1}P_{n}}$, and $d_{1,i}^{⊥}=\sqrt{s_{1,i}^{⊥}}$, the projection on the q−1 dimensional space orthogonal to this axis. Projections ${\bar{d}}_{1,i}$, with 1<i≤n, give the first column of X in Equation (2) and the recursive analysis of matrix $D^{\prime }$ with $D_{i,j}^{\prime }=s_{i,j}^{⊥}$ would provide the q−1 remaining columns of this matrix.

Formally, ${\bar{d}}_{1,i}$ is

(6) $${\bar{d}}_{1,i}=d_{1,i}\;\cos\alpha$$

which, by the cosine rule becomes

(7) $${\bar{d}}_{1,i}=\frac{1}{2\;d_{1,n}}\;\left(s_{1,i}+s_{1,n}-s_{i,n}\right)$$

The square of the projection in the subspace orthogonal to the vector pointing from $P_{1}$ to $P_{n}$ is

(8) $$\begin{array}{cc} s_{1,i}^{⊥} & =s_{1,i}-{\bar{d}}_{1,i}^{2}=s_{1,i}-\frac{1}{4\;s_{1,n}}\;{\left(s_{1,i}+s_{1,n}-s_{i,n}\right)}^{2} \end{array}$$

The interval evaluation of Equations (7) and (8) would produce overestimation [21] since, for instance, $s_{1,n}=d_{1,n}^{2}$ and, thus, $d_{1,n}$ appears twice in them. Tighter estimations can be obtained using the monotonicity analysis introduced in [15], which we recall next.

If a function is monotone in a given domain, it is clear that its extrema are the boundary of the domain. Moreover, if the domain is an axis-aligned box, as it is our case, we can appeal the following well-known proposition to find exact bounds without overestimation:

**Proposition** **1.**  [65]*. Let $x=(x_{1},\cdots ,x_{q})$ be a tuple of q real interval-valued variables defining an axis-aligned box such that $x_{i}\in \left[x_{i}^{-},x_{i}^{+}\right]$, and let $f\left(x\right)\in \left[f^{-},f^{+}\right]$. If f is continuous and locally monotonic with respect to each argument, then*

$$f^{-}=\min_{x\in H}f\left(x\right)$$

*and*

$$f^{+}=\max_{x\in H}f\left(x\right)$$

*where H is the set of $2^{q}$ vertices of the box defined by* **x***.*

**Figure 4:**
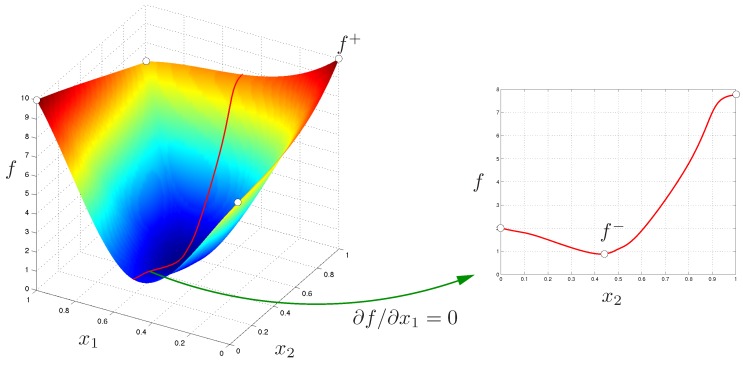
Schematic illustration of how to determine the exact bounds of $f(x_{1},x_{2})$ analyzing its monotonicity. We have to consider the value of the function in selected corners of the domain and the sub-spaces where $\partial f/\partial x_{1}=0$ or where $\partial f/\partial x_{2}=0$, if such conditions may hold in the domain. These sub-spaces have to be recursively analyzed. For instance, the plot at the right shows this analysis for the first case. The evaluation of *f* on $x_{1}^{-}=0$ and $x_{2}^{+}=1$ gives $f^{+}$, the upper bound of this function in the prescribed domain. In this example, the lower bound $f^{-}$ is obtained in the analysis of the sub-space where $\partial f/\partial x_{1}=0$. Figure adapted from [15].

If the derivatives of *f* with respect to $x_{1},\cdots ,x_{n}$ are know, then the vertices defining the extrema can be identified without the need of evaluating *f* in all of them: the maximum would correspond to the vertex where, for i=1…n, $x_{i}$ is $x_{i}^{-}$ if $\partial f/\partial x_{i}<0$ and $x_{i}^{+}$ otherwise. The minimum would be in a vertex defined with the opposite criterion. Therefore, the analysis of the sign of $\partial f/\partial x_{i}$ reveals the monotonic domains for *f* as a function of $x_{i}$. Moreover, the boundaries between monotonic domains, i.e., the points where the derivative is 0, have to be analyzed separately, considering also the monotonicity, since they may include the extrema of *f*, as illustrated in Figure 4.

Applying this monotonicity analysis to ${\bar{d}}_{1,i}$ as defined in Equation (7), we obtain

(9) $$\begin{array}{cc} \frac{\partial {\bar{d}}_{1,i}}{\partial d_{1,i}} & =\frac{d_{1,i}}{d_{1,n}} \end{array}$$

(10) $$\begin{array}{cc} \frac{\partial {\bar{d}}_{1,i}}{\partial d_{i,n}} & =-\frac{d_{i,n}}{d_{1,n}} \end{array}$$

(11) $$\begin{array}{cc} \frac{\partial {\bar{d}}_{1,i}}{\partial d_{1,n}} & =1-\frac{{\bar{d}}_{1,i}}{d_{1,n}} \end{array}$$

**Figure 5:**
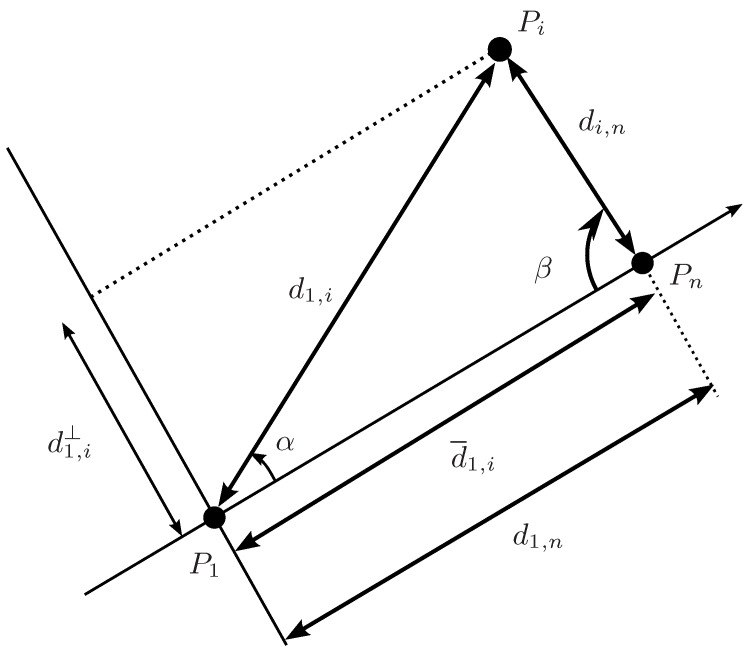
Special situation where $\bar{P_{1}P_{n}}$ is orthogonal to $\bar{P_{i}P_{n}}$. This case has to be considered separately in the monotonicity analysis of ${\bar{d}}_{1,i}$.

The first two derivatives are positive and negative, respectively, and thus the maximum of ${\bar{d}}_{1,i}$ can only be at $d_{1,i}=d_{1,i}^{+}$ and $d_{i,n}=d_{i,n}^{-}$ and the minimum at $d_{1,i}=d_{1,i}^{-}$ and $d_{i,n}=d_{i,n}^{+}$. If Equation (11) is also monotone, only the corresponding limit, $d_{1,n}=d_{1,n}^{-}$ or $d_{1,n}=d_{1,n}^{+}$, need to be considered. However, if the ranges of ${\bar{d}}_{1,i}$ and $d_{1,n}$ intersect, Equation (11) could vanish and the extrema of ${\bar{d}}_{1,i}$ can be in the subspace where ${\bar{d}}_{1,i}=d_{1,n}$, which must be analyzed separately. In this situation, shown in Figure 5, $\bar{P_{1}P_{n}}$ is orthogonal to $\bar{P_{i}P_{n}}$, and thus

(12) $${\bar{d}}_{1,i}=\sqrt{d_{1,i}^{2}-d_{i,n}^{2}}$$

which is monotone in both $d_{1,i}$ and $d_{i,n}$. Therefore, the maximum of ${\bar{d}}_{1,i}$ in Equation (12) is at $d_{1,i}=d_{1,i}^{+}$ and $d_{i,n}=d_{i,n}^{-}$ and the minimum at $d_{1,i}=d_{1,i}^{-}$ and $d_{i,n}=d_{i,n}^{+}$. Thus, if $\bar{P_{1}P_{n}}$ can be orthogonal to $\bar{P_{i}P_{n}}$, these points must be considered as potential extrema of ${\bar{d}}_{1,i}$.

The monotonicity analysis for $s_{1,i}^{⊥}$ as defined in Equation (8) requires computing

(13) $$\begin{array}{cc} \frac{\partial s_{1,i}^{⊥}}{\partial d_{1,i}} & =2\;d_{1,i}\;\left(1-\frac{{\bar{d}}_{1,i}}{d_{1,n}}\right) \end{array}$$

(14) $$\begin{array}{cc} \frac{\partial s_{i,n}^{⊥}}{\partial d_{i,n}} & =\frac{2\;d_{1,i}\;{\bar{d}}_{1,i}}{d_{1,n}} \end{array}$$

(15) $$\begin{array}{cc} \frac{\partial s_{1,i}^{⊥}}{\partial d_{1,n}} & =-2\;{\bar{d}}_{1,i}\;\left(1-\frac{{\bar{d}}_{1,i}}{d_{1,n}}\right) \end{array}$$

These three derivatives are monotone, except for ${\bar{d}}_{1,i}=0$ or ${\bar{d}}_{1,i}=d_{1,n}$. In the first case, we have that

(16) $$s_{1,i}^{⊥}=s_{i,n}-s_{1,n}$$

and, in the second one,

(17) $$s_{1,i}^{⊥}=s_{1,i}-s_{1,n}$$

Both expressions are linear and, thus, monotone.

A similar monotonicity analysis can be done for ${\bar{d}}_{i,j}={\bar{d}}_{1,i}-{\bar{d}}_{1,j}$, with 1<i<j≤n, and for the corresponding $s_{i,j}^{⊥}$ to obtain tight ranges for all of them.

### 4.2. Range Reduction

When the projection process is repeated q−1 times, the problem becomes one-dimensional and all ranges should correspond to distances between points on a line. Then, the congruence between the ranges can be enforced using the triangular equality [66]. However, in our case, orientation constraints can be exploited to further reduce the ranges, as detailed in Algorithm 1. This algorithm is a variant of the Floyd-Warshall method to determine the shortest paths between all pairs of nodes in a weighted graph [5]. The distance between two points, $P_{i}$ and $P_{k}$, is the shortest possible via any intermediate point $P_{j}$. Since distances are not signed, when considering a triplet $P_{i}$, $P_{k}$, and $P_{j}$, we have to consider the case where vectors $\vec{P_{j}P_{i}}$ and $\vec{P_{j}P_{k}}$ have the same or opposite orientations on the line (see Figure 1). This gives two possible values for $d_{i,k}$, $d_{i,k}^{-}$ and $d_{i,k}^{+}$ (see lines 5 and 9 in Algorithm 1) and the range for this distance must be set to the interval hull of both of them, i.e., the smallest interval containing the two given solution ranges. Function HULL in line 12 of Algorithm 1 implements this procedure. Note that HULL(a,b)=HULL(b,a) and that, by convention, HULL(a,∅)=a.

If $\sigma_{F,i,k}$ with $F=\{P_{i_{1}},\ldots ,P_{i_{q}}\}$ and $i_{1}=1$, is set to 1 or −1, and the original problem is projected using the axes defined by $\bar{P_{i_{1}}P_{i_{2}}},\ldots ,\bar{P_{i_{1}}P_{i_{q}}}$, the orientation of the triplet $P_{1}$, $P_{i}$, and $P_{k}$ is fixed, i.e., $\sigma_{1,i,k}$ in the projected problem has the same value as $\sigma_{F,i,k}$ in the original problem. This is illustrated in Figure 1, where the relative orientation of the tetrahedra formed by $\left\{P_{j},P_{l},P_{m},P_{i}\right\}$, and $\left\{P_{j},P_{l},P_{m},P_{k}\right\}$ translates to a particular orientation of vector $\vec{P_{j}^{\prime \prime }P_{i}^{\prime \prime }}$ with respect to vector $\vec{P_{j}^{\prime \prime }P_{k}^{\prime \prime }}$. This can be exploited to consider only one of the two solutions of the triangular equality. Since all the possible combinations of projections are iteratively used in the algorithm, all the orientations fixed in the original problem are eventually used when reducing the ranges.

**Figure 6:**
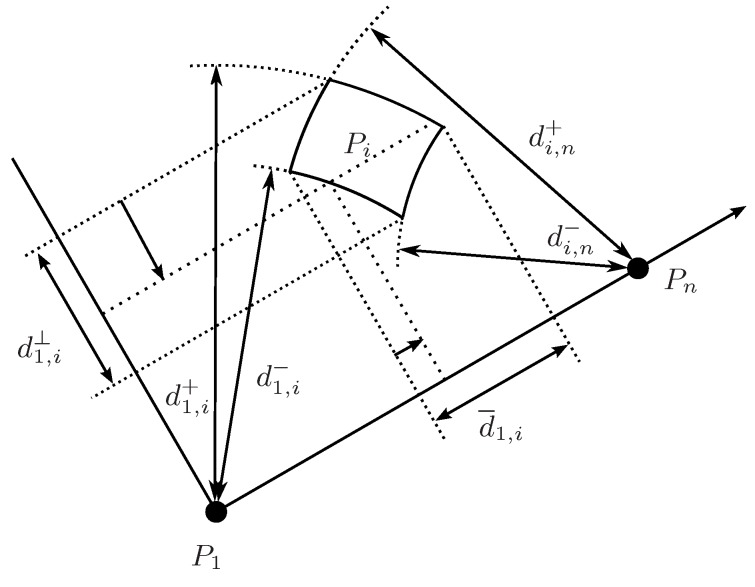
Possible location of $P_{i}$ for the given ranges of $d_{1,i}$ and $d_{i,n}$ and the corresponding projections on the axis defined by $\bar{P_{1}P_{n}}$, ${\bar{d}}_{1,i}$, and on its orthogonal complement, $d_{1,i}^{⊥}$. A reduction in the range of $d_{1,i}^{⊥}$ can be translated into a reduction of the range of ${\bar{d}}_{1,i}$.

The reduction in the orthogonal subspace can be translated to the projection axis defined by $\bar{P_{1}P_{n}}$ taking into account that, as shown in Figure 6, $P_{i}$ must be at distance $d_{1,i}\in \left[d_{1,i}^{-},d_{1,i}^{+}\right]$ from $P_{1}$ and at distance $d_{i,n}\in \left[d_{i,n}^{-},d_{i,n}^{+}\right]$ from $P_{n}$, i.e.,

(18) $$\begin{array}{cc} s_{1,i} & ={\bar{d}}_{1,i}^{2}+s_{1,i}^{⊥} \end{array}$$

(19) $$\begin{array}{cc} s_{i,n} & ={\left({\bar{d}}_{1,i}-d_{1,n}\right)}^{2}+s_{1,i}^{⊥} \end{array}$$

Thus,

(20) $${\bar{d}}_{1,i}=HULL\left({\bar{d}}_{1,i}\cap \sqrt{s_{1,i}-s_{1,i}^{⊥}},{\bar{d}}_{1,i}\cap \sqrt{s_{i,n}-s_{1,i}^{⊥}}+d_{1,n}\right)$$

Once the ranges for ${\bar{d}}_{1,i}$ with 1<i<n are clipped, the triangular equality can be used to propagate the reductions to the other projections on the axis defined by $\bar{P_{1}P_{n}}$.

**Algorithm 1:** Range reduction using distance and orientation constraints.  **ReduceRange**(*D*)  **input**: The matrix of squared distances  **output**: The reduced matrix of squared distances. 
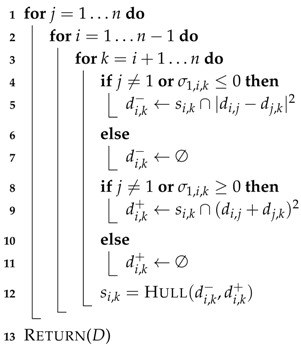


### 4.3. Back-Projection

The reduced ranges on the projected sub-spaces can be back-projected to recover possibly reduced distance ranges in the original space taking into account that, as shown in Figure 3,

(21) $$s_{1,i}={\bar{d}}_{1,i}^{2}+s_{1,i}^{⊥}$$

and

(22) $$\begin{array}{cc} s_{1,i} & =s_{1,n}+s_{i,n}-2\;d_{1,n}\;d_{i,n}\;\cos\beta =s_{1,n}+s_{i,n}-2\;d_{1,n}\;{\bar{d}}_{i,n} \end{array}$$

The expression in Equation (21) is monotone and, for Equation (22), we have

(23) $$\begin{array}{cc} \frac{\partial s_{1,i}}{\partial d_{i,n}} & =2\;d_{i,n} \end{array}$$

(24) $$\begin{array}{cc} \frac{\partial s_{1,i}}{\partial {\bar{d}}_{i,n}} & =-2\;d_{1,n} \end{array}$$

(25) $$\begin{array}{cc} \frac{\partial s_{1,i}}{\partial d_{1,n}} & =2\;d_{1,n}-2\;{\bar{d}}_{i,n} \end{array}$$

that are monotone except for ${\bar{d}}_{i,n}=d_{1,n}$. In this case

(26) $$s_{1,i}=s_{i,n}-s_{1,n}$$

which is monotone both in $s_{i,n}$ and in $s_{1,n}$.

**Figure 7:**
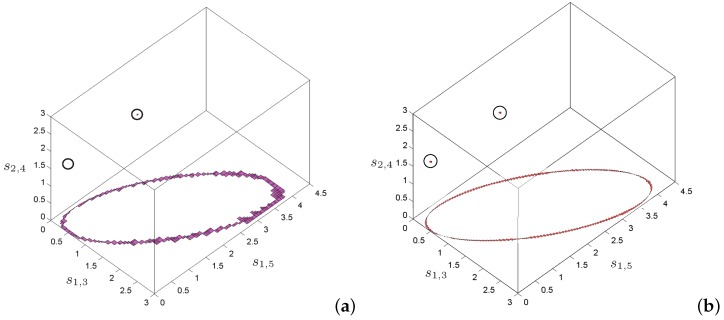
Approximation of the solution space for a problem with two isolated solutions and a one-dimensional flex. The isolated solutions are encircled to make them visible. (**a**) Using a projection and back-projection relying on standard interval arithmetics we obtain a rough approximation of the flex. (**b**) Using the monotonicity-based alternative introduced in this paper over-estimations are avoided and the flex is tightly bounded.

The improvement of the projection and back-projection procedures based on the monotonicity analysis presented in this paper with respect to those presented in [21] based on interval arithmetics are illustrated in Figure 7 for a particular example. The figure shows the accuracy in the approximation of the solution set for a problem with two isolated solutions and a one-dimensional flex. The approach using standard interval-based projection and back-projection correctly isolates the solutions, but, for the flex, the approximation is rough and irregular. With the same parameters, the projection and back-projection methods based on the monotonicity analysis generate a more regular and accurate approximation. This example also illustrates that the proposed approach can deal with problems even if they include flex ambiguities, which are hardly considered in previous works.

## 5. Experiments

To illustrate the localization method introduced in this paper, we apply it to solve the localization of a fleet of robots carrying sensors to survey a flat environment and the position analysis of a sensor moved by a parallel manipulator. A non-optimized and non-parallelized Matlab implementation of the approach introduced in this paper including the examples described next is attached to this paper as multimedia material.

### 5.1. Localization of Networked Mobile Robots

Networked mobile robots interact over a signal exchange network for its coordinated operation and behavior. Such systems have applications in diverse areas of science and engineering such as rescue operations [67], networked vehicles [68] or distributed arrays of sensors [69].

Robot teams can exploit collaboration to maintain global positioning as they move through space. Each robot can be equipped, for instance, with an infrared sensor and a camera to estimate its distance to the teammates [70]. Moreover, the camera provides information about the relative bearing of the robot with respect to the rest of the team. Thus, the global positioning of the team must fulfill a set of distance and orientation constraints as the ones given in Section 3.

Since the distance measurements are noisy, or simply missing due to the limited range of the infrared sensors, maximum-likelihood estimators that determine the most probable position of all the robots have been used in the past [71]. Nevertheless, it is difficult to give realistic probability density functions of the sensor readings due to the complexity of the physical process on which the estimated distance is based. Instead, if we simply assume that errors in measurements are bounded, it is possible to apply a Distance Geometry approach to obtain tight bounds, as exemplified next.

**Figure 8:**
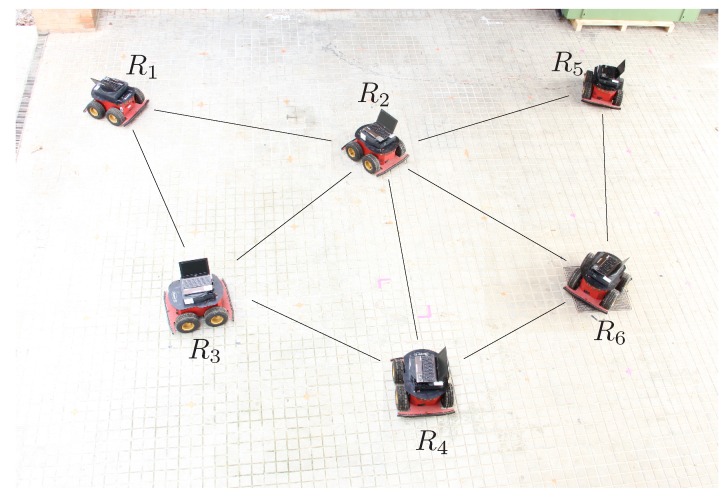
A fleet or robots. Each robot is equipped with an infrared sensor to measure the distances to nearby teammates. The lines in the figure represent the distances actually measured. The relative orientation of the triangles is given by cameras mounted on the robots.

**Figure 9:**
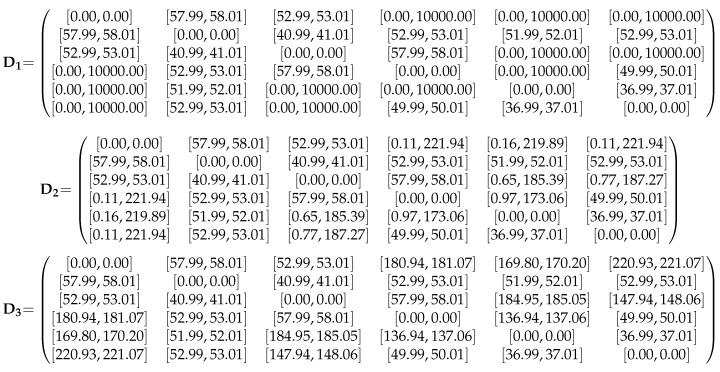
$D_{1}$ stands for the matrix of input ranges as provided by the infrared sensors; $D_{2}$, for the matrix of ranges after imposing triangular inequalities; and $D_{3}$, the matrix of ranges resulting from applying the method introduced in this paper.

Consider the team of six mobile robots shown in Figure 8. The maximum range of the infrared sensors is 8 meters and distances are estimated with a 3% of error. Moreover, the orientations of the triangles given by the robot’s cameras are those in the figure. For instance, $\sigma_{F,1,4}$ with $F=\{P_{2},P_{3}\}$ is +1. In these conditions, the sensors provide the matrix $D_{1}$ of distance ranges between the robots shown in Figure 9. Some of the distances are not measured due to sensor problems, or because the robots are further than the maximum span of the infrared sensor. Thus, for those distances we can only assume that they in the range [0,+∞]. In the actual implementation 100 meters is used as the maximum possible distance since this is larger than the sum of all the measured distances in the problem.

**Figure 10:**
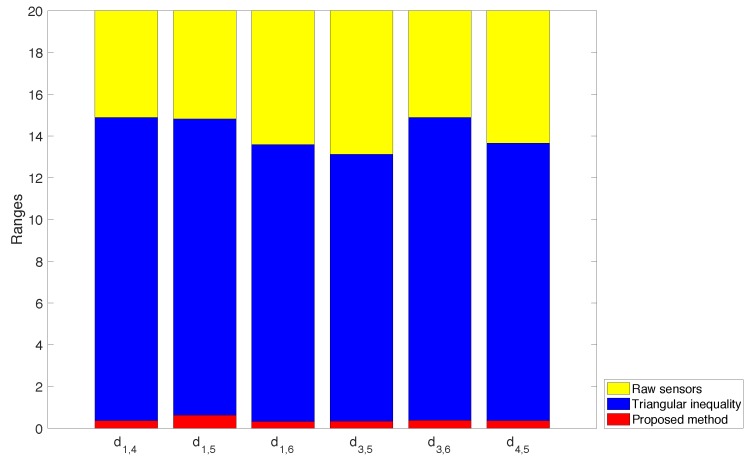
The reduction of the size of the intervals for the non-measured distances in the problem when applying alternative tightening procedures.

After applying the triangular inequalities, i.e., the standard tool for range reduction when using Distance Geometry, the undefined ranges get bounded, as shown in matrix $D_{2}$. Due to the ambiguities inherent to the used distance formulation, this process only produces a trivial reduction of some of the ranges. In contrast, applying the method introduced in this paper, matrix $D_{3}$ is obtained, where some of the ranges are significantly narrower than the ones obtained with the standard method. For instance $s_{1,4}$ is reduced from [0.11,221.94] to [180.94,181.07]. The plot in Figure 10 shows a graphical representation of the reduction of the ranges when we apply alternative tightening processes.

Although not intended for this case, the proposed algorithm can be applied to the same problem, but without orientation constraints. Then, the problem has 8 solutions corresponding to the different orientations of the triangles sharing segments $\bar{R_{2}R_{3}}$, $\bar{R_{2}R_{4}}$, and $\bar{R_{2}R_{6}}$. The procedure introduced in this paper isolates these eight solutions without generating any empty box, i.e., boxes resulting from a bisection process that do not include any solution point. Such empty boxes indicate inefficiencies in the range reduction procedures. Existing branch-and-bound approaches operating in distance space [19,21] isolate the same 8 solutions, but they generate more empty boxes in the process because such approaches rely on interval arithmetics which introduce overestimations. Moreover, there is no way to incorporate orientation constraints in these solvers to directly isolate one of the solutions.

Finally, the approach proposed in this paper may have some resemblance to the MDS method, although MDS projects distance matrices, while we implicitly project points in Cartesian space using distance information and MDS assumes a probabilistic model of the errors while we adopt a set theoretic one. Therefore, the results from both approaches are significantly different. The MDS method generates an initial distance matrix sampling in the ranges given by the sensors and approximating the rest of distances by the shortest path on the graph defined by the sampled values. The initial distance matrix is improved projecting it to the closest point in the least square sense in the cone of semidefinite matrices. In the projection, all the distances in the matrix are modified and, thus, there is no way to ensure that the result is included in the ranges provided by the sensors. Consequently, this procedure has a low probability of generating any of the actual solutions of the problem. Actually, in our tests, none out of 100 executions of the MDS procedure returned any of the 8 valid solution for the problem, which are correctly identified by the procedure introduced in this paper.

### 5.2. Position Analysis of a Parallel Robot

Parallel manipulators, if well calibrated, can move a sensing device with a high position and angular accuracy [72]. The octahedral parallel manipulator considered next was studied, for instance, in [73] where a closed formula for its forward position analysis is provided. However, the analytical approaches do not take into account orientation constraints such as the ones considered in this paper. The particular robot analyzed has equilateral base and platform with side 12 and 6 distance units, respectively and the leg lengths are

$$\begin{array}{ccccc} d_{1,4}=19.8 & & d_{2,4}=18 & & d_{2,5}=18\\ d_{3,5}=17 & & d_{3,6}=14.9 & & d_{1,6}=17.8 \end{array}$$

with these values, the platform can be positioned in six different poses with respect to the robot’s base.

**Figure 11:**
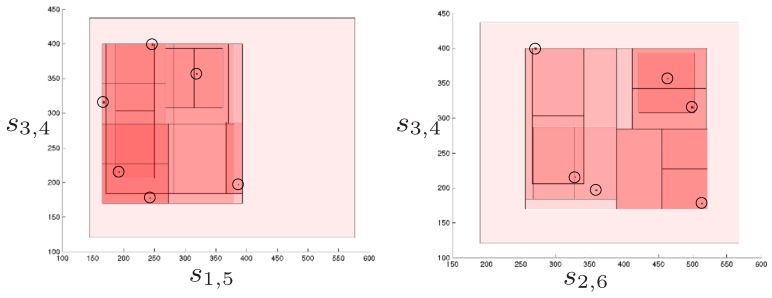
Two views of the boxes processed to isolate the six valid poses of the octahedral manipulator. The solutions are the encircled tiny boxes. The initial box, i.e., the lighter red box, is reduced and split until the six solutions are properly isolated. It can be appreciated that only few of the processed boxes include no solution, i.e., they are empty boxes.

Considering that this problem is three-dimensional and, thus, two consecutive projections are necessary before reducing the ranges, 150 different combinations of projection are possible. With these projections, the solutions of the position analysis are isolated after processing 23 boxes: six are solutions, six are empty boxes (i.e, boxes with no solution inside), and 11 boxes are internal nodes of the search tree. Despite the double projection introduces dependencies that are not considered when applying them as independent operations, the performance is remarkably good since very few empty boxes are generated, in comparison with the number of solutions. Figure 11 shows two views of the boxes processed by the solver when isolating the six solutions.

**Figure 12:**
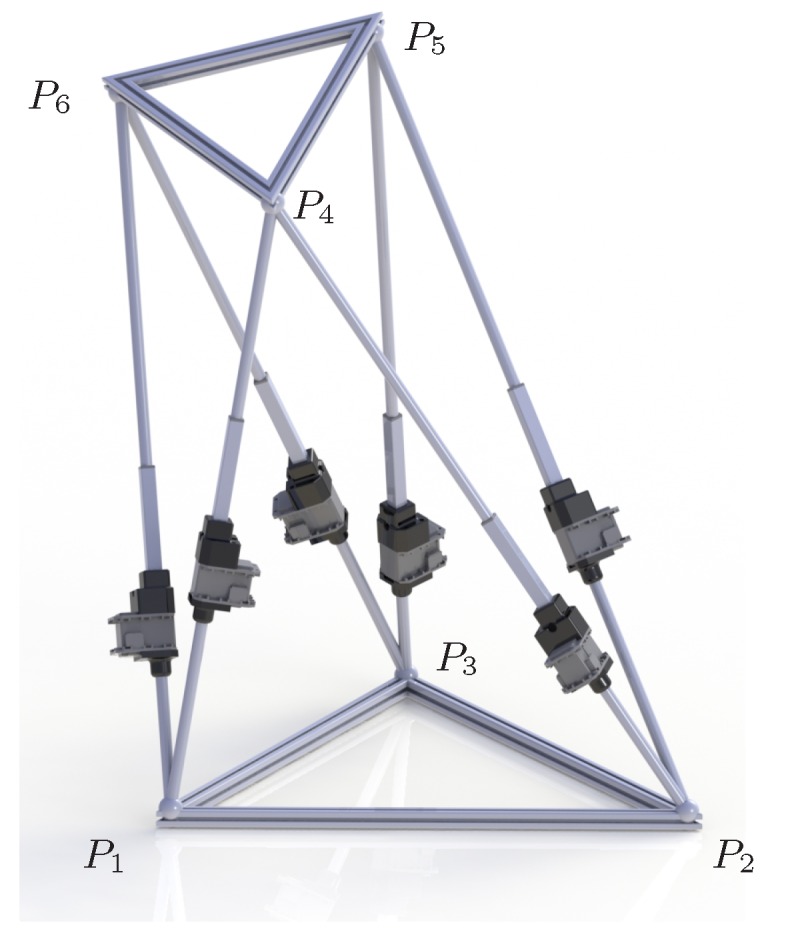
Solution of the direct kinematics of the octahedral manipulator obtained when considering orientation constraints.

**Figure 13:**
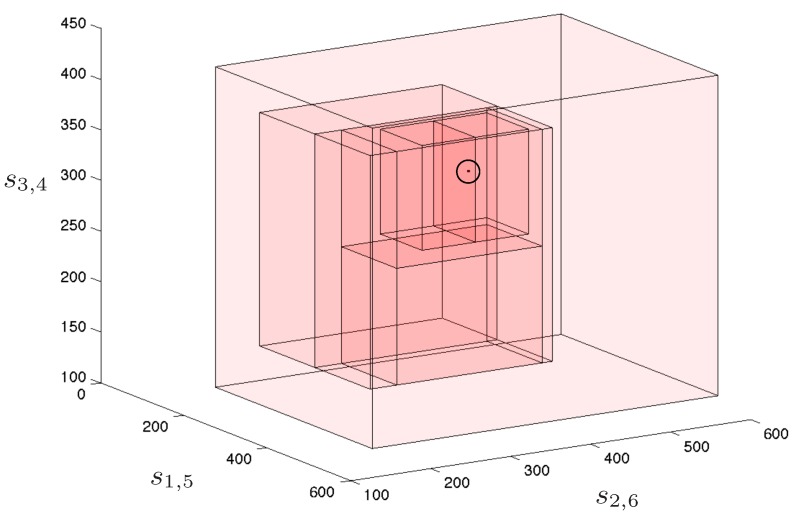
Boxes processed when introducing orientation constraints in the direct kinematics problem for the octahedral manipulator. The solution is the encircled tiny box.

However, the six solutions are not equal taking into account aspects such as leg interference and the stiffness of the platform. If the following orientation constraints are added to the problem $\sigma_{1,2,4,5,6}=-1$, $\sigma_{2,4,5,1,3}=-1$, $\sigma_{2,3,5,4,6}=-1$, $\sigma_{3,5,6,1,2}=-1$, $\sigma_{1,3,6,4,5}=-1$, $\sigma_{1,4,6,2,3}=-1$, only the solution shown in Figure 12, which is the preferred configuration in practice, is valid. This solution is isolated after processing 7 boxes (1 solution, 3 empty, and 3 intermediate boxes). These boxes are represented in Figure 13. The solver effectively takes advantage of the orientation constraints since the number of processed boxes significantly reduces when taking them into account. Previous approaches either analytical or numerical would produce the same result with or without orientation constraints since they only consider them in a post-process stage.

## 6. Conclusions

This paper extends a projection and back-projection method for solving systems of distance constraints to consider orientation constraints as well, and provides new procedures for its three basic steps: the projection, the range reduction, and the back-projection. The novel projection and back-projections are based on a monotonicity analysis that avoids the overestimations introduced when using standard interval arithmetics. The proposed procedures give an effective solver that is conceptually simple and whose basic operations can be easily parallelized. This opens a wide range of applications for Distance Geometry in fields such as Robotics, structural biology, nano-technology, or network sensors.

The presented experiments prove the feasibility of the proposed method, but it can be improved in many directions. For instance, several (combinations of) projections can be used in each problem, but not all of them contribute equally to the range reduction. Thus, it is our objective to further analyze the projection procedure to characterize the best projections.

## Supplementary Materials

The following are available online at http://www.mdpi.com/1424-8220/16/7/1096/s1. The Matlab implementation of the method presented in this paper is available as a supplementary material attached to this paper.
